# Penetrating Ballistic Brain Injury Produces Acute Alterations in Sleep and Circadian-Related Genes in the Rodent Cortex: A Preliminary Study

**DOI:** 10.3389/fneur.2021.745330

**Published:** 2021-10-21

**Authors:** Andrea Mountney, Jennifer Blaze, Zhaoyu Wang, Michelle Umali, William Jesse Flerlage, Jacqueline Dougherty, Yongchao Ge, Deborah Shear, Fatemeh Haghighi

**Affiliations:** ^1^Walter Reed Army Institute of Research (WRAIR), Silver Spring, MD, United States; ^2^Department of Neuroscience, Icahn School of Medicine at Mount Sinai, New York, NY, United States; ^3^Friedman Brain Institute, Icahn School of Medicine at Mount Sinai, New York, NY, United States; ^4^Research and Development Service, James J. Peters Veterans Affairs Medical Center, Bronx, NY, United States

**Keywords:** traumatic brain injury, circadian, sleep, prefrontal cortex, gene expression

## Abstract

Traumatic brain injury (TBI) affects millions of Americans each year, with extremely high prevalence in the Veteran community, and sleep disturbance is one of the most commonly reported symptoms. Reduction in the quality and amount of sleep can negatively impact recovery and result in a wide range of behavioral and physiological symptoms, such as impaired cognition, mood and anxiety disorders, and cardiovascular effects. Thus, to improve long-term patient outcomes and develop novel treatments, it is essential to understand the molecular mechanisms involved in sleep disturbance following TBI. In this effort, we performed transcriptional profiling in an established rodent model of penetrating ballistic brain injury (PBBI) in conjunction with continuous sleep/wake EEG/EMG recording of the first 24 h after injury. Rats subjected to PBBI showed profound differences in sleep architecture. Injured animals spent significantly more time in slow wave sleep and less time in REM sleep compared to sham control animals. To identify PBBI-related transcriptional differences, we then performed transcriptome-wide gene expression profiling at 24 h post-injury, which identified a vast array of immune- related genes differentially expressed in the injured cortex as well as sleep-related genes. Further, transcriptional changes associated with total time spent in various sleep stages were identified. Such molecular changes may underlie the pathology and symptoms that emerge following TBI, including neurodegeneration, sleep disturbance, and mood disorders.

## Introduction

The Centers for Disease Control and Prevention estimated in 2010 that 2.5 million emergency department visits were related to traumatic brain injuries (1). TBIs of varying severity can result from numerous causes such as motor vehicle accidents, sports related blows to the head, closed-head injury, penetrating ballistic injury, and exposure to an explosive blast (2). In the US Armed Forces, TBI remains prevalent, with over 430,000 members of the military being diagnosed between 2000 and 2021 (3). For patients, their families, and communities, the consequences of TBI extend far beyond the event that caused injury. Acute and long-term sequelae of severe TBI include impaired motor function and diminished cognitive ability. In addition, behavioral changes, sleep-wake disturbances (4, 5), and psychiatric disorders often occur. These effects can result in chronic disability and possibly an increased risk for neurodegenerative disorders (4, 6, 7) with patients requiring a high degree of care. With the rising number of individuals sustaining severe TBI, understanding the molecular circuitry involved in both the acute and long-term sequelae are critically important to the health and productivity of these patients. Specifically, understanding the acute changes to brain function and behavior that take place immediately following TBI allow for future studies investigating how these translate to long-lasting outcomes.

Sleep complaints are highly prevalent in individuals with a history of TBI (8–11). Sleep disturbance, often in the form of insomnia, can be chronic and persist years after the initial TBI was sustained. However, acute changes in sleep have also been measured and reported in the clinical population following TBI (12). Commonly reported sleep disturbances following TBI include problems falling and remaining asleep, overall poor sleep quality, and early awakening (8). The fatigue following non-restorative sleep can severely impair day time functioning and affect mood (9). Chronic sleep disturbance can exacerbate psychiatric symptoms resulting from the TBI itself and has been significantly associated with increased anxiety (13) and suicidal ideation (14). In addition to insomnia, major sleep disorders have been reported in individuals with severe TBI, with diagnoses including insomnia, post-traumatic hypersomnia, delayed sleep phase syndrome, irregular sleep-wake pattern disorder, and periodic limb movement disorder (5, 8–11). Further, studies have identified alterations in vigilance states following TBI using EEG recording, including changes to rapid eye movement (REM) sleep, non-REM (NREM) sleep, and wakefulness following TBI in the clinical population (15).

A number of animal models of TBI have been developed to study TBI-related pathology (including sleep) that employ (1) weight drops from varying heights (16, 17), (2) exposure to blast overpressure (18), (3) controlled cortical impact (CCI) (19), (4) fluid percussion injury (20), and (5) penetration by ballistic projectiles (21–23). In a penetrating brain injury, a high velocity projectile penetrates the skull and forms a temporary cavity in the brain that may be several times the size of the projectile (23–27). Penetrating injuries can result from not only gunshot wounds but also from shrapnel released at high velocity from an explosive blast. While the number of studies of penetrating brain injuries has been limited, our group has established and extensively characterized an animal model of a non-fatal penetrating ballistic-like blast injury (PBBI) that produces symptoms similar to those observed in humans. We have observed blood brain barrier damage, edema, neuroinflammation, and cell death following PBBI in rodents (25, 26, 28, 29). In addition, our use of a rodent model has allowed for extensive functional and behavioral testing, which has revealed injury-related cognitive and motor impairments (29–31). The pathological changes in brain-wave activity have been extensively characterized in the PBBI model. Long-term continuous electroencephalography (cEEG) studies in PBBI-injured rodents have revealed periodic epileptiform discharges, increased slow-wave activity, and non-convulsive seizures, comparable to human TBI patients, that respond positively to early intervention with anti-epileptic therapeutics (32–36). While there has been thorough testing of emotional, cognitive, and EEG deficits following PBBI in rodents, to our knowledge no studies have characterized acute sleep-wake disturbances in this model.

We have previously reported epigenetic and transcriptional changes in a rodent model of mild TBI resulting from low-level repeated blast overpressure exposure (37, 38). Of particular relevance to our investigation of PBBI-related sleep disruption, we found blast-exposure dependent changes in DNA methylation and gene expression at various sleep-associated gene loci, including *Aanat, Homer1a, Nos1, Chrna3, Cacna1b*, and *Per3* (37, 38). To our knowledge, there have been no studies that have investigated transcriptome-wide changes in the cortex in response to PBBI specifically, but the studies discussed as well as our own have identified the brain's transcriptional machinery as a key component of the brain's response to traumatic injury that may be related to sleep disturbances following TBI. To build upon these findings, in this study we assessed acute changes in sleep-wake activity, a symptom often reported by TBI patients (39), and associated transcriptional changes in the cortex acutely following a well-established animal model of PBBI. The state of the art *in vivo* sleep recordings and next-generation sequencing approaches implemented in our study has allowed discovery of acutely appearing behavioral and molecular perturbations that may lay the groundwork for the neurologic and psychiatric symptoms following TBI in the clinical population.

## Methods

### Animals

**Figure 1:**
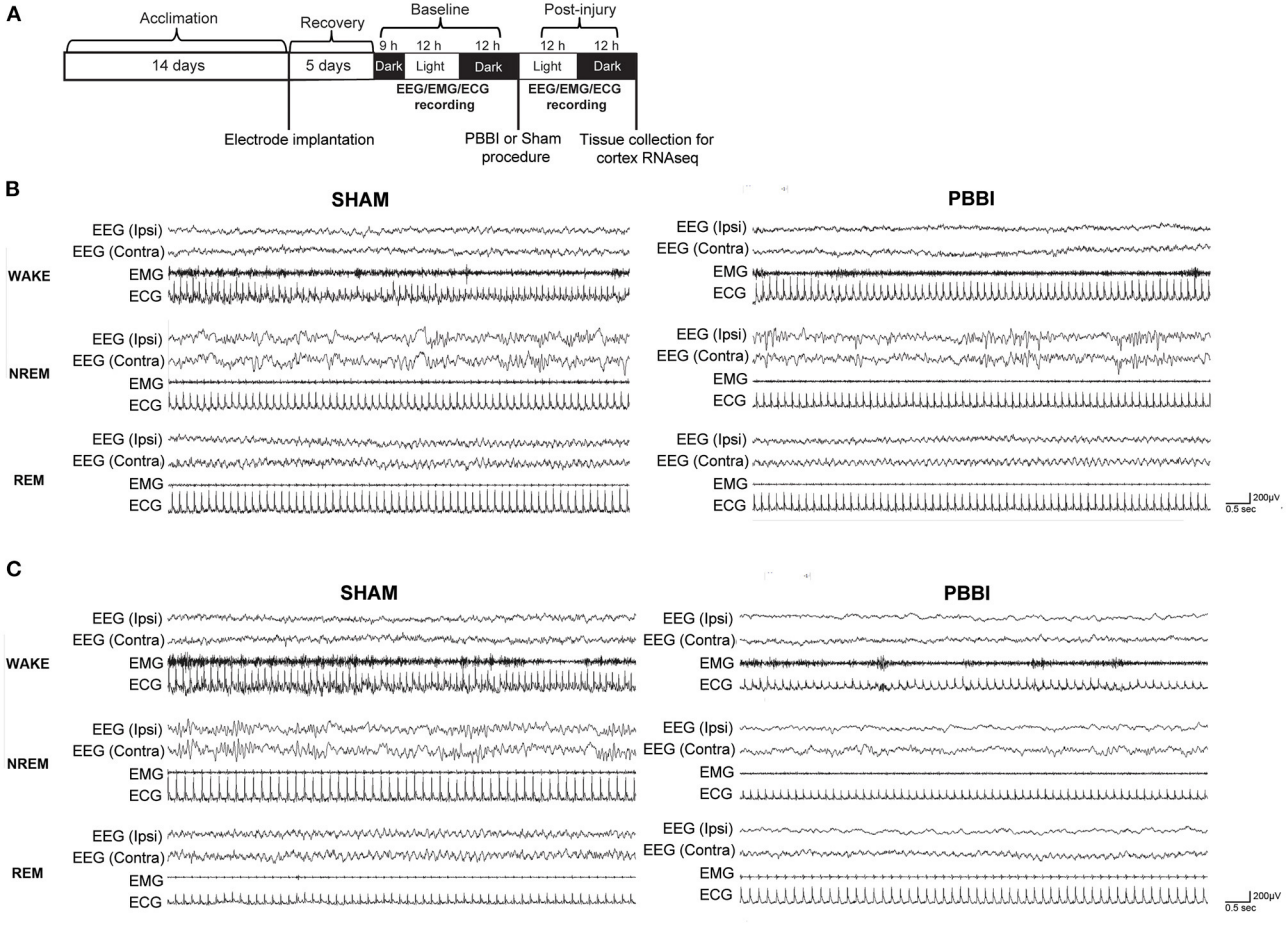
**(A)** Schematic of experimental timeline for EEG/EMG/ECG recording and molecular analysis. Representative recordings **(B)** pre-injury and **(C)** acute (<24 h) post-PBBI in sham and PBBI rats. PBBI was performed on the right (ipsilateral) side. Spectral tracings from all three vigilance states (wake, NREM, and REM) following PBBI demonstrated slightly attenuated voltages and reduced frequencies acutely over the ipsilateral cortex. No overt changes over the left (contralateral) cortex were detected. EMG and ECG tracings demonstrate muscle activity and heartbeat, respectively.

Subjects included 12 adult male Sprague-Dawley rats (275–320 g, Charles River Labs, Raleigh, NC) that were randomized to PBBI (*n* = 6) or sham (*n* = 6) groups. Animals were maintained on a 12:12 light:dark cycle with lights on at 0,900 h (Zeitgeber time; ZT0) and off at 2,100 h (ZT12) for at least 2 weeks prior to surgery. All animals had access to food and water *ad libitum*. All animal housing, injury, and sleep-wake assessment procedures were performed at Walter Reed Army Institute of Research (WRAIR) in an American Association for Accreditation of Laboratory Animal Care International (AAALACi) accredited facility under an Institutional Animal Care and Use Committee (IACUC) approved protocol, in compliance with the Animal Welfare Act and other federal statutes and regulations relating to animals and experiments involving animals and adhered to principles stated in the Guide for the Care and Use of Laboratory Animals. For all surgical procedures, anesthesia was induced by 5% isoflurane and maintained at 2% isoflurane delivered in oxygen with a body temperature maintained at 37.0°C using a heating blanket (Harvard Apparatus, Holliston, MA). A schematic timeline of experimental procedures is presented in Figure 1A. A power analysis set to a desired power level of 0.8 with alpha level of 0.05 was conducted using SigmaPlot 13 (Systat Software Inc., San Jose, CA) to determine the group sample size. One sham rat was excluded after it prematurely detached the recording electrode prior to the experimental endpoint. Final cohort numbers were as follows: PBBI - 6, Sham - 5.

### EEG/EMG Electrode Implantation Surgery

Implantation surgeries were performed on groups of four animals per week to accommodate the availability of sleep monitoring cages. All surgeries were performed as Category E in order to mitigate analgesia-induced effects on sleep recordings. The electrode configuration for sleep recordings was modified from previously published cEEG recording methods (34–36). For sleep recordings, rats were anesthetized and surgically implanted with four stainless steel screws (0.3175 cm, Component Supply Company, Fort Mead, FL) through bilateral burr holes over the frontal and parietal skull regions (1 mm anterior and 4 mm posterior to bregma, ±3.0 mm lateral to midline) in a stereotaxic frame. Two additional electrodes were implanted posterior to lambda over the transverse sinus to serve as reference and ground. Each screw was soldered to insulated stainless steel wire electrodes (Plastics One, 0.25 mm in diameter). The exposed skull screws and wires were secured in place with dental cement (Butler Schein, Dublin, OH). An additional set of leads was placed bilaterally in the nuchal muscles for EMG recording and one ECG electrode was placed over the upper left abdomen. The free end of each electrode wire was tunneled subcutaneously and soldered to a Dale multi-pin connector (March Electronics, West Hempstead, NY). Implantation surgeries took approximately 1 h to perform and rats were allowed to recover individually and acclimate in singly-housed Plexiglas monitoring cages for a minimum of 5 days before recording.

### Penetrating Ballistic Blast Injury (PBBI)

PBBI and sham surgeries were performed on groups of *n* = 4 animals per week to accommodate the availability of sleep monitoring cages. All surgeries were performed as Category E in order to mitigate analgesia-induced effects on sleep recordings. Following a recovery period of approximately 5 days after EEG/implantation, animals (*n* = 4) were removed from sleep monitoring cages and randomized and balanced between PBBI and sham groups. All surgeries were conducted on the same day in the early morning (between ZT23 and ZT0 – dark period) to ensure the shortest interval between surgery and the start of EEG/EMG data collection. PBBI animals received a right unilateral frontal PBBI as previously described (24, 31). This penetrating injury was a model of a shockwave with a ballistic component that created a brain cavity. Animals were anesthetized with 2% vapor isoflurane and positioned in a stereotaxic frame. To administer the PBBI, we employed a computer-controlled simulated ballistic injury device (Mitre Corp, McLean VA) with an attached custom-designed 20 G stainless steel tubular probe. Along one end of this probe were fixed holes covered by airtight elastic tubing. Following unilateral (right) frontal pole craniectomy (+4.5 mm anteroposterior, +2 mm medio-lateral from bregma), the probe was inserted at an angle of 50° from the vertical axis and 25° counter-clockwise from the anterior-posterior axis to a distance of 1.2 cm from the dural surface of the brain. An automated pulse-generator was used to quickly (<40 ms) inflate/deflate the elastic tubing into an elliptical balloon to a size roughly equal to 10% brain volume. The PBBI procedure took approximately 10 min to complete. Sham animals were subjected to identical procedures, including equal time under anesthesia, but without probe insertion.

### Sleep-Wake Assessment

Continuous EEG/EMG data were collected at WRAIR from animals individually housed in custom-designed (24 x 18 inch) Plexiglass recording chambers equipped with multichannel gold contact swivel communicators (Dragonfly Inc., Ridgeley, WV) for cEEG monitoring. A flexible shielded cable connected the swivel to the multipin connector on the rat skull to allow free movement of the animal for the duration of the experiment. The swivel commutator was interfaced with an EEG amplifier, and EEG/EMG/ECG signals were continuously monitored and recorded at a sampling frequency of 400 Hz using a computerized Grass Technologies LTM Monitoring amplifier and data acquisition system (Natus Neurology Incorporated - Grass Products, Warwick, RI). EEG and EMG signals were used to objectively classify 1 s epochs into vigilance states (wakefulness, slow-wave sleep, and REM sleep) using a computerized algorithm based on EEG and EMG amplitudes and frequencies as well as the probability that the subject will transition from a given stage to another (Neuroscore 3.0 software, Data Sciences International, St Paul, MN) by investigators blinded to experimental groups. The sleep score for a given 10 s period was based on the summary of 1 s epochs. Epochs were assigned to a specific vigilance state when <50% of the epoch fulfilled the criteria for a sleep stage. Continuous recordings were conducted for 33 h pre-injury (baseline) and subsequently for 24 h post-Injury or sham procedure.

Wakefulness periods were characterized by fast, low voltage unsynchronized EEGs associated with high voltage EMG (muscle tone). NREM sleep (Slow-wave sleep, SWS) was characterized by high voltage, low frequency EEG associated with low amplitude EMG. REM sleep was defined on the basis of low voltage unsynchronized EEG activity in the theta range (6–9 Hz) associated with low voltage EMG and absence of activity. Data could not be obtained while rats were undergoing experimental manipulations.

The time spent in vigilance states (minutes/hour) was calculated for 33 h baseline and 24 h post-injury recordings. For individual sleep metrics, all 10 s epochs were summed into 60 or 720-min bins, differentiated between light and dark periods, and group-averaged to examine the amount of time spent in vigilance states of wake, NREM, and REM sleep. Polysomnograms of each hemisphere (contralateral and ipsilateral to injury) were scored separately. Sleep-wake parameters used in these analyses included duration in vigilance states, bout analysis (number and duration), sleep state transitions, Wake after sleep onset, WASO (defined duration of wakefulness after initial sleep onset), and latency (onset) to NREM or REM sleep (defined as the time interval to the first six consecutive NREM or REM 10 s epochs), and duration of sleep/wake states.

To assess changes in spectral power between sham and PBBI rats, quantitative EEG relative power spectra from ipsilateral and contralateral cortical electrodes were computed for 10 s epochs during 24 h periods during baseline and post-injury/sham procedure from 0.5 to 24 Hz using a Fast Fourier transform (FFT) with a Hamming window and overlap ratio of 0.5. Total power Fast Fourier transforms (FFT) were calculated in 10 s epoch using Neuroscore software with 256 bin size and a Hann (cosine-bell) data window using spectral data extracted from each epoch. Epochs with software-identified artifacts were excluded. FFTs were sorted by vigilance state and hemisphere and average EEG power data were displayed according to the following power bands: delta (0.5–4 Hz), theta (5–8 Hz), alpha (9–12 Hz), sigma (13–16 Hz), and beta (17–24 Hz). Data for each subject were expressed as a percentage of baseline. One PBBI animal was excluded from REM period qEEG analysis, as it did not exhibit post-injury REM sleep.

Descriptive statistics were averaged within experimental groups and expressed as mean ± SEM in defined time intervals. Statistical analysis of differences in sleep parameters and changes in power spectra were determined by repeated two-way analysis of variance (ANOVA) or two-way ANOVA when appropriate followed by a Bonferroni correction for multiple comparisons using SigmaPlot 13 (Systat Software Inc., San Jose, CA) and GraphPad Prism 6 (GraphPad Software, La Jolla, CA). A P ≤ 0.05 was considered statistically significant.

### RNA Isolation

After completion of the EEG/EMG recordings, animals were sacrificed. Brains were harvested, underwent sectioning (40), and cortical tissue samples were stored in a −80°C freezer. DNA and RNA were isolated simultaneously from tissue samples using the Zymo ZR-Duet™ DNA/RNA MiniPrep (Zymo Research Group, Irvine, CA) as per the manufacturer's instructions, and stored in −80°C freezer till further processing.

### Transcriptional Profiling via RNA-seq

RNA-sequencing and analysis were performed at the NY Genome Center. RNA sequencing libraries were prepared using the KAPA Stranded RNA-Seq Kit with RiboErase (Kapa Biosystems) in accordance with the manufacturer's instructions and sequenced on an Illumina HiSeq2500 sequencer (v4 chemistry) using 2 x 50 bp cycles.

Alignment was done with STAR 2.4.2a (41). STAR index was made using splice junction annotation database for guidance, both genome sequence and annotation files of Ensembl Rnor 6.0. Quantification of gene expression was done using featureCounts 1.4.3-p1 (42) with Ensembl Rnor 6.0 annotation.

Differential expression analyses looking at the effect of injury and sleep stage duration were performed with the Bioconductor package DESeq2 (version 1.14.1) (43). The analysis was performed for each side of the brain separately. DESeq2 allows for tests of differential expression by use of negative binomial generalized linear models. Dispersion estimates and logarithmic fold changes incorporate data-driven prior distributions. In addition to using DESeq2 to identify changes in gene expression dependent on injury status, we also used DESeq2 to identify genes with expression correlated with total time spent in each sleep stage regardless of injury: for each sleep measurement (wakefulness, NREM, and REM sleep), we tested for differential expression between samples using the sleep measurement as a covariate in the design model. Multiple-test corrected *p*-values (adjusted p) were used to identify differentially expressed genes. In addition, DESeq2 provided transformations for count data that stabilized variance across the mean. The regularized-logarithm transformation (rlog) gave results similar to the standard log2 transformation of normalized counts for those genes with high counts. For lower count genes, the rlog transformation shrank values toward, the gene's averages across all samples. An empirical Bayesian prior was used on sample differences in the form of a ridge penalty such that the rlog-transformed data neared homoscedasticity, allowing for direct determination of distances between samples. To explore the relationship between sleep parameters and gene expression in both brain hemispheres, correlation coefficients were correlated with total REM duration, total NREM, and total wake. Directionality of the correlation between transcription and sleep are indicated as +corr (positive correlation) or –corr (negative correlation) (see Figure 8). Pathway analysis was performed with StringDB and by Ingenuity Pathway Analysis software (IPA®, Qiagen, Redwood City, www.qiagen.com/ingenuity).

## Results

### Alterations in Sleep Architecture Associated With PBBI

Bilateral recordings from Sham and PBBI-injured groups were collected and scored into the three vigilance states of wakefulness, NREM, and REM sleep. Prior to injury, EEG recordings from both hemispheres were qualitatively similar in all vigilance states in both Sham and PBBI groups (Figure 1B). Visual inspection of post-injury recordings suggested PBBI rats exhibited an acute attenuation of EEG signal amplitude and reductions in EEG frequencies, which were more salient over the ipsilateral (injured) hemisphere compared to baseline recordings (Figure 1C).

**Figure 2:**
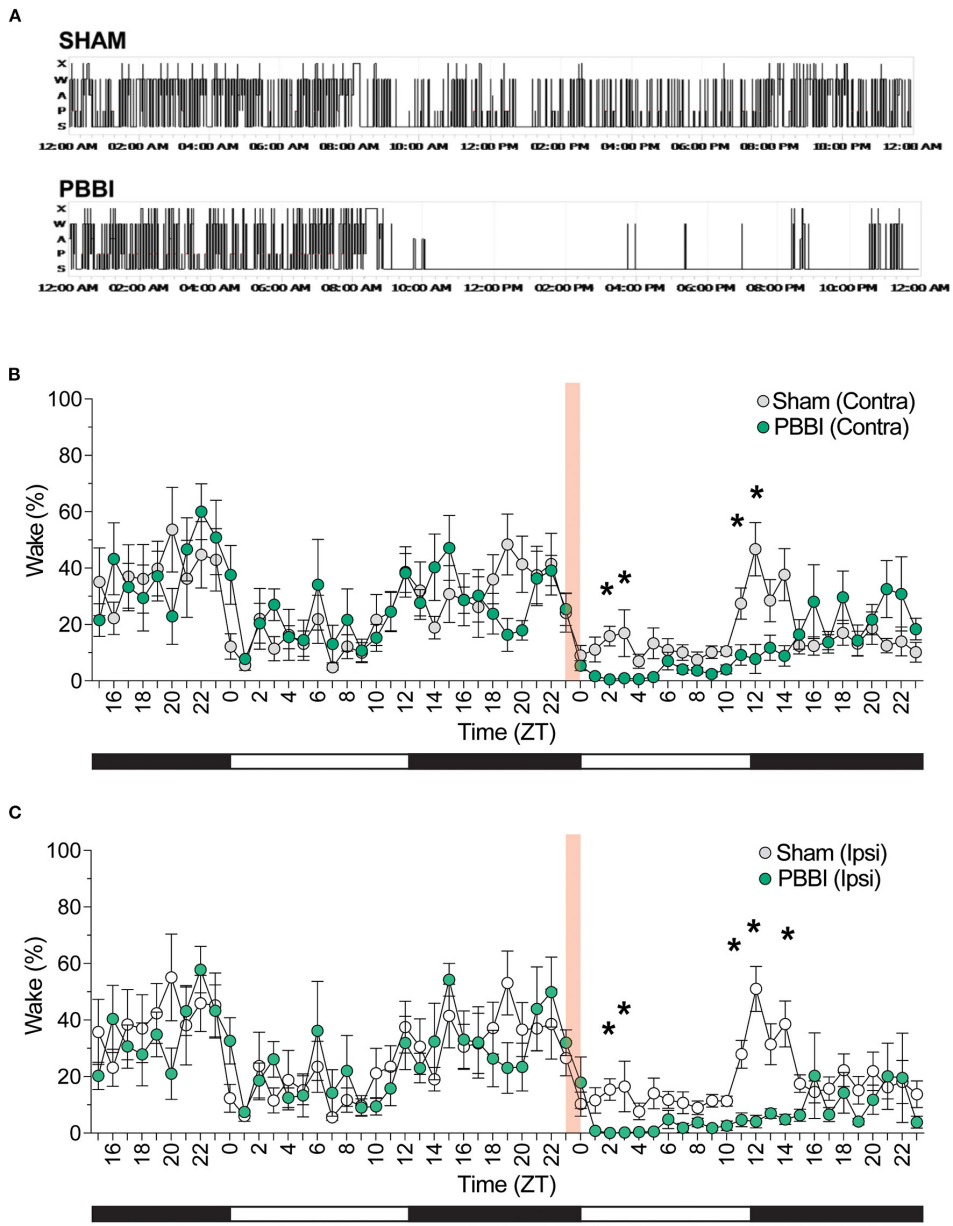
PBBI-induced acute alterations in sleep–wake architecture. **(A)** Representative hypnograms (24 h) of sham and injured rats during baseline through 12 h post-injury. **(B)** Sleep was analyzed for 33 h prior to injury and EEG recordings were scored into vigilance states and grouped in 1 h bins showing for **(B)** contralateral and **(C)** ipsilateral hemispheres of injured rats and sham animals. Red shaded block denotes time of injury. Shaded and unshaded bars indicate dark and light periods of activity. All data are depicted as the mean ± SEM. Comparison between PBBI and sham performed by repeated measures two-way ANOVA with Bonferroni *post-hoc* test. *N* = 6 PBBI/ 5 sham. Significant changes were more prevalent on the ipsilateral side (*p < 0.05).

**Figure 3:**
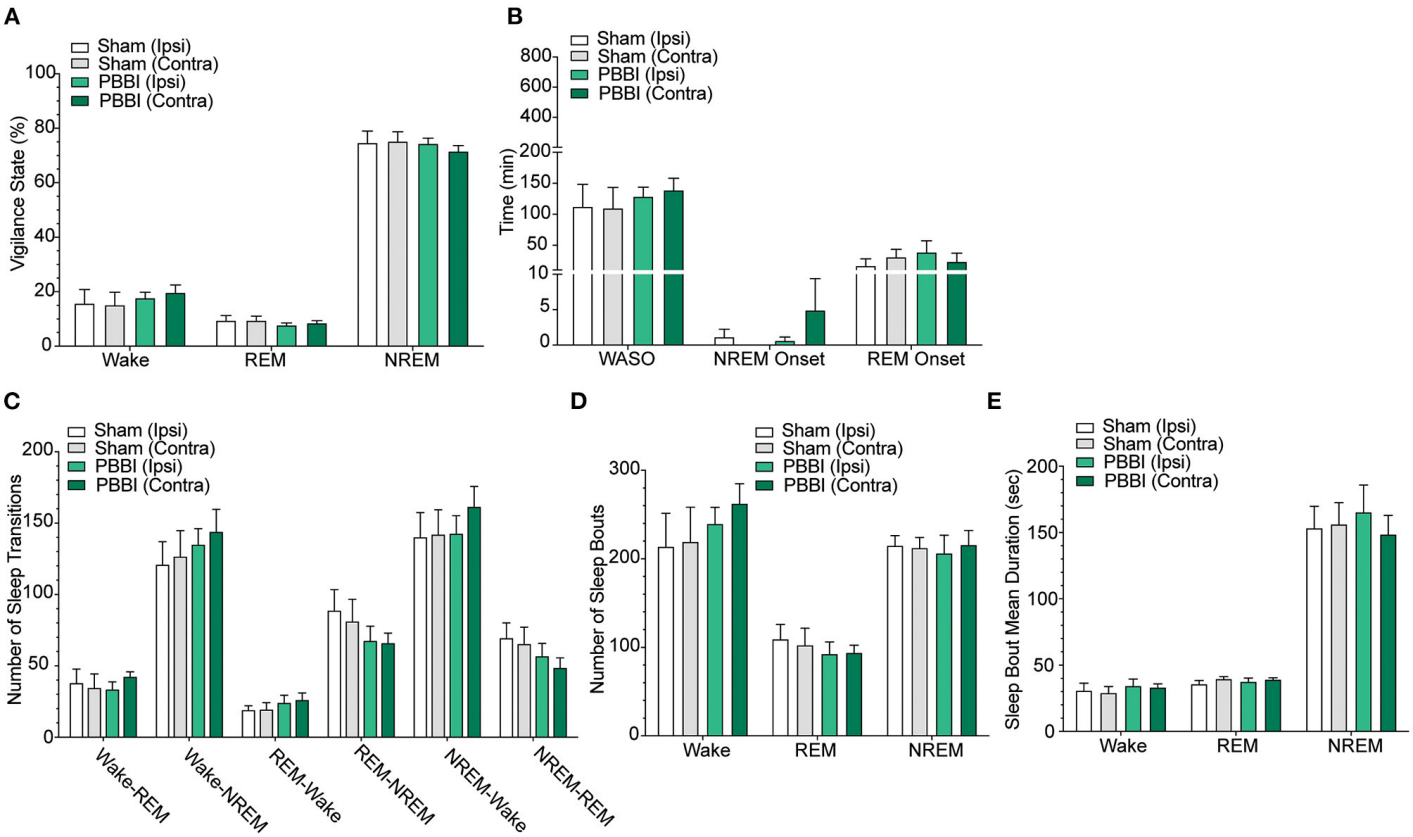
Sleep parameters for contralateral and ipsilateral hemispheres in sham and PBBI animals in the light pre-injury baseline phase. Time spent in each vigilance state was collapsed over 12 h bins during baseline recordings and compared between hemispheres and injury groups. There were no significant differences between hemisphere and injury groups across **(A)** vigilance states, **(B)** sleep parameters, **(C)** quantity of sleep stage transitions, **(D)** number of sleep stage bouts, **(E)** mean bout duration. Time spent in each vigilance state was collapsed over 12h bins and compared between hemispheres and injury groups. *N* = 6 PBBI / 5 sham. All data are depicted as the mean ± SEM. WASO = wake after sleep onset.

Quantitative sleep-wake staging indicated significant differences in sleep architecture between experimental cohorts across a number of sleep parameters–representative hypnograms for injured and sham animals are shown in Figure 2A. Prior to injury, both cohorts showed similar baseline sleep architecture across both hemispheres: animals displayed light-dependent rhythmic changes which oscillated between sleep-wake states in accordance with light and dark phases (Figures 2B, 3; Supplementary Table 1); however, within 2 h post-injury, PBBI rats displayed significant decreases in wakefulness which were most salient at 11, 12, and 14 h post-injury (Figures 2B,C).

**Figure 4:**
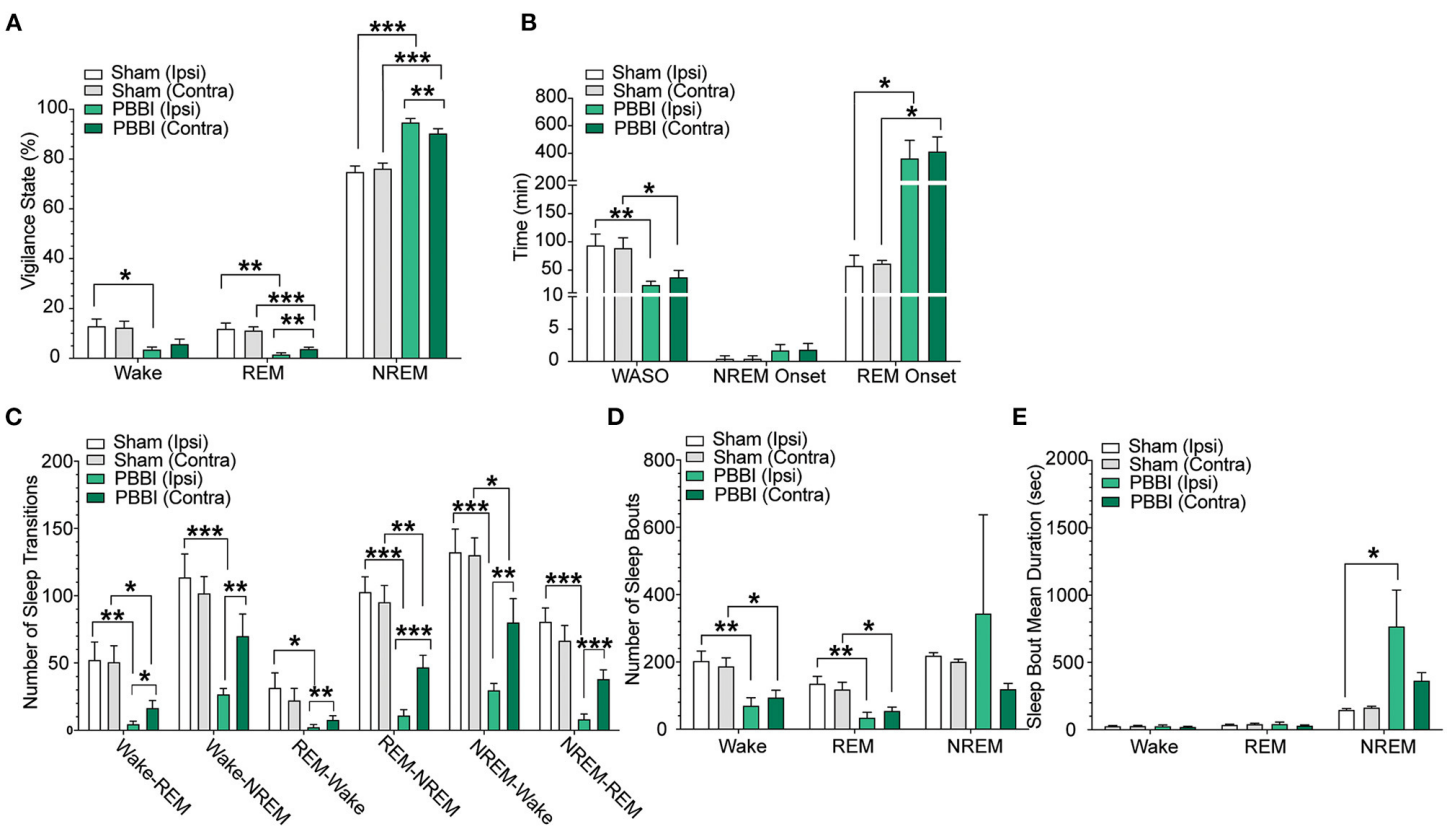
Sleep parameters for contralateral and ipsilateral hemispheres in sham and PBBI animals in the light phase during the acute post-injury period. Time spent in each vigilance state was collapsed over 12 h bins and compared between hemispheres and injury groups. **(A)** PBBI rats showed significant increases in NREM sleep with accompanying decreases in REM sleep, **(B)** shorter periods of wake after sleep onset (WASO), **(C)** fewer sleep stage transitions, **(D)** fewer sleep stage bouts, and **(E)** increased mean bout duration during NREM sleep (ipsilateral side only). Significant changes were more prevalent on the ipsilateral side. N=6 PBBI / 5 sham. All data are depicted as the mean ± SEM. WASO: wake after sleep onset (***p < 0.001, **p < 0.01, *p < 0.05).

**Figure 5:**
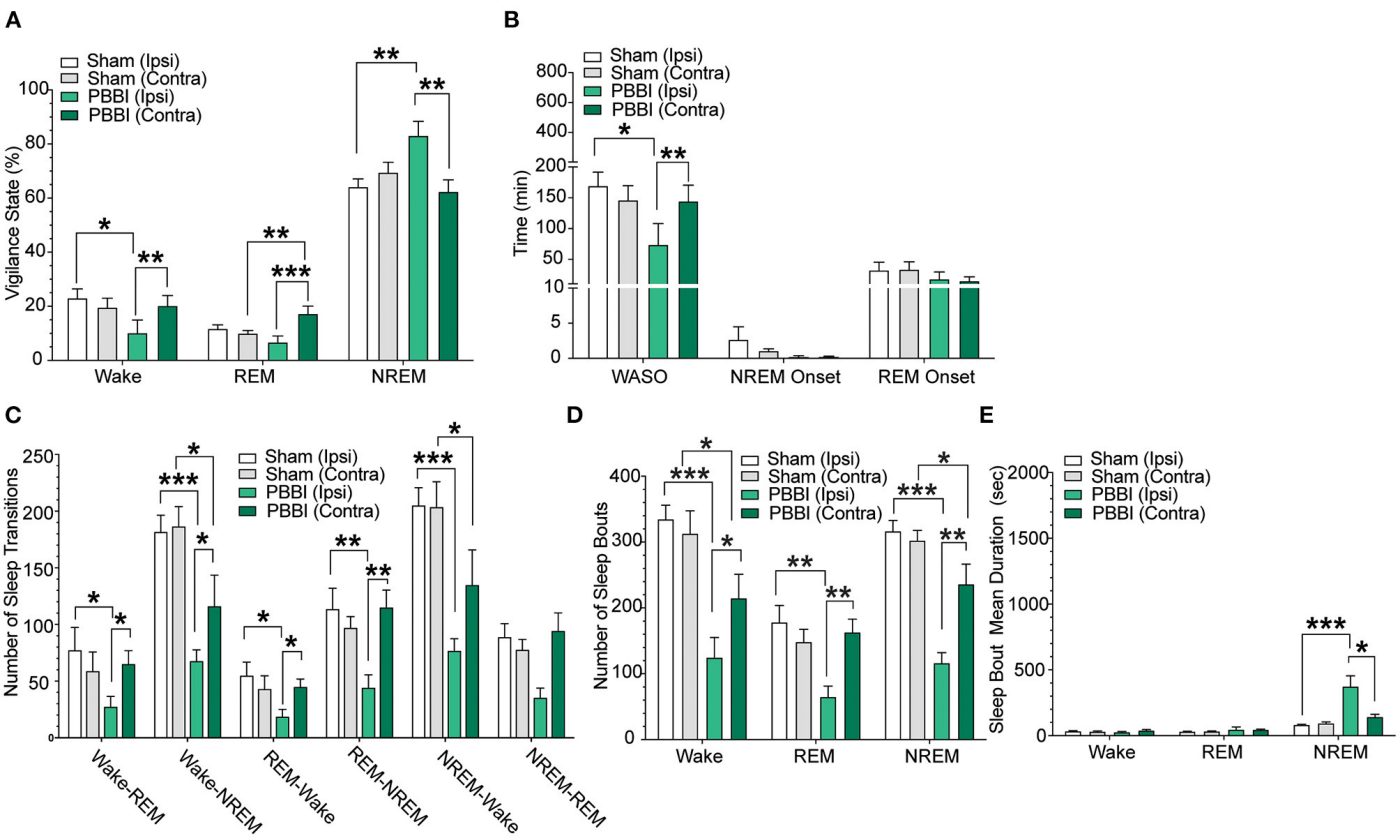
Sleep parameters for contralateral and ipsilateral hemispheres in sham and PBBI animals in the dark phase during the acute post-injury period. Time spent in each vigilance state was collapsed over 12 h bins and compared between hemispheres and injury groups. **(A)** PBBI rats showed significant increases in NREM sleep on the ipsilateral hemisphere only. **(B)** WASO, NREM onset, and REM onset were not significantly different between sham and injury groups. PBBI rats showed significant decreases in the number of **(C)** sleep transitions and **(D)** sleep bouts and increased NREM mean bout duration **(E)**. Significant changes were more prevalent on the ipsilateral side. N=6 PBBI / 5 sham. All data are depicted as the mean ± SEM. WASO = wake after sleep onset (***p < 0.001, **p < 0.01, *p < 0.05).

In order to characterize post-injury sleep changes, we assessed vigilance stages and sleep parameters during both light and dark phases. Importantly, baseline pre-injury sleep metrics were not significantly different between PBBI and sham cohorts (Figures 3A–E; Supplementary Table 1). In contrast, PBBI rats displayed significant changes across multiple sleep metrics during both light and dark periods. During the light phase, PBBI rats showed an increased sleep propensity with increases in NREM sleep and concomitant reductions in wakefulness, REM sleep, WASO and delayed REM onset (Figures 4A,B; Supplementary Table 2). Significant changes were more salient on the injured side. Since TBI can alter the capacity to sustain wakefulness, we evaluated changes in the number of sleep transitions and bouts between sleep stages throughout the sleep-wake cycle. During the post-injury light phase, PBBI rats showed significantly fewer sleep transitions between all three vigilance states (Figure 4C; Supplementary Table 2). Reductions in sleep transitions were accompanied by significantly fewer bouts of wakefulness and REM sleep, compared to sham counterparts (Figure 4D; Supplementary Table 2). Although PBBI rats did not display significant differences in the number of NREM sleep bouts, the average duration in injured animals was over 4 times longer than those detected in sham animals, and was evident only in recordings from the injured side (Figure 4E; Supplementary Table 2). Analysis of sleep parameters during the subsequent dark (active) phase, showed similar sleep architecture changes in PBBI rats: increases in NREM sleep, decreased wakefulness, fewer sleep stage transitions, fewer sleep bouts, and increased NREM bout durations, which were more evident from recordings on the injured side (Figures 5A-E; Supplementary Table 3).

**Figure 6:**
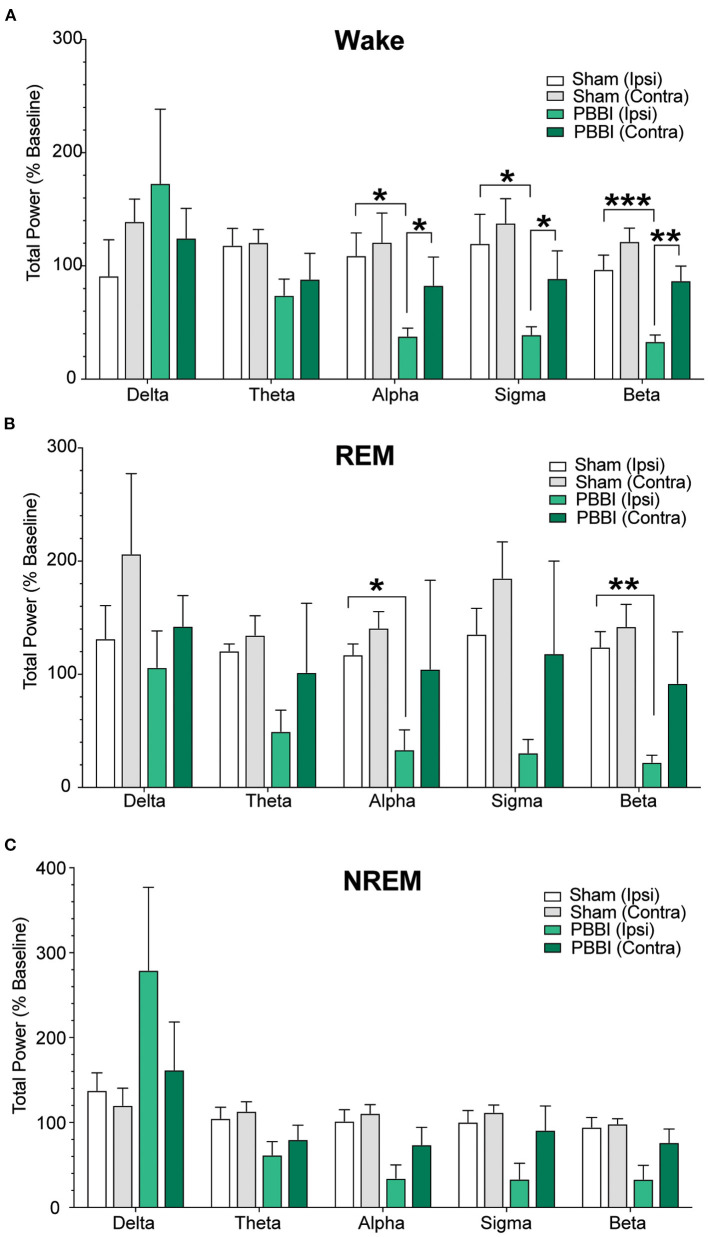
Comparison of relative total power between PBBI and sham animals. Power data were extracted from baseline and post-injury periods from both hemispheres and binned into frequency bands and sorted according to vigilance states: **(A)** wake, **(B)** REM, and **(C)** NREM sleep. PBBI rats showed significant reductions in relative power at higher frequencies with trends toward increased delta power. N = 5 PBBI / 5 sham. All data are depicted as the percentage of baseline mean ± SEM (***p < 0.001, **p < 0.01, *p < 0.05).

In order to examine the quality of NREM and REM sleep following brain injury, we assessed post-injury spectral changes in both hemispheres. During periods of wakefulness and NREM sleep, PBBI rats showed significant decreases in higher frequency bands (alpha through beta) and trended toward increases in delta power compared to sham. Notably, these differences were only detected in cEEG recordings from the injured side. Sham rats did not show significant inter-hemispheric differences in power spectra across multiple frequency bands during all three vigilance states (delta, theta, alpha, sigma, and beta). Spectral analysis during periods of REM sleep was not significantly different between groups (Figures 6A-C; Supplementary Table 4).

### Gene Expression Changes Associated With PBBI

**Figure 7:**
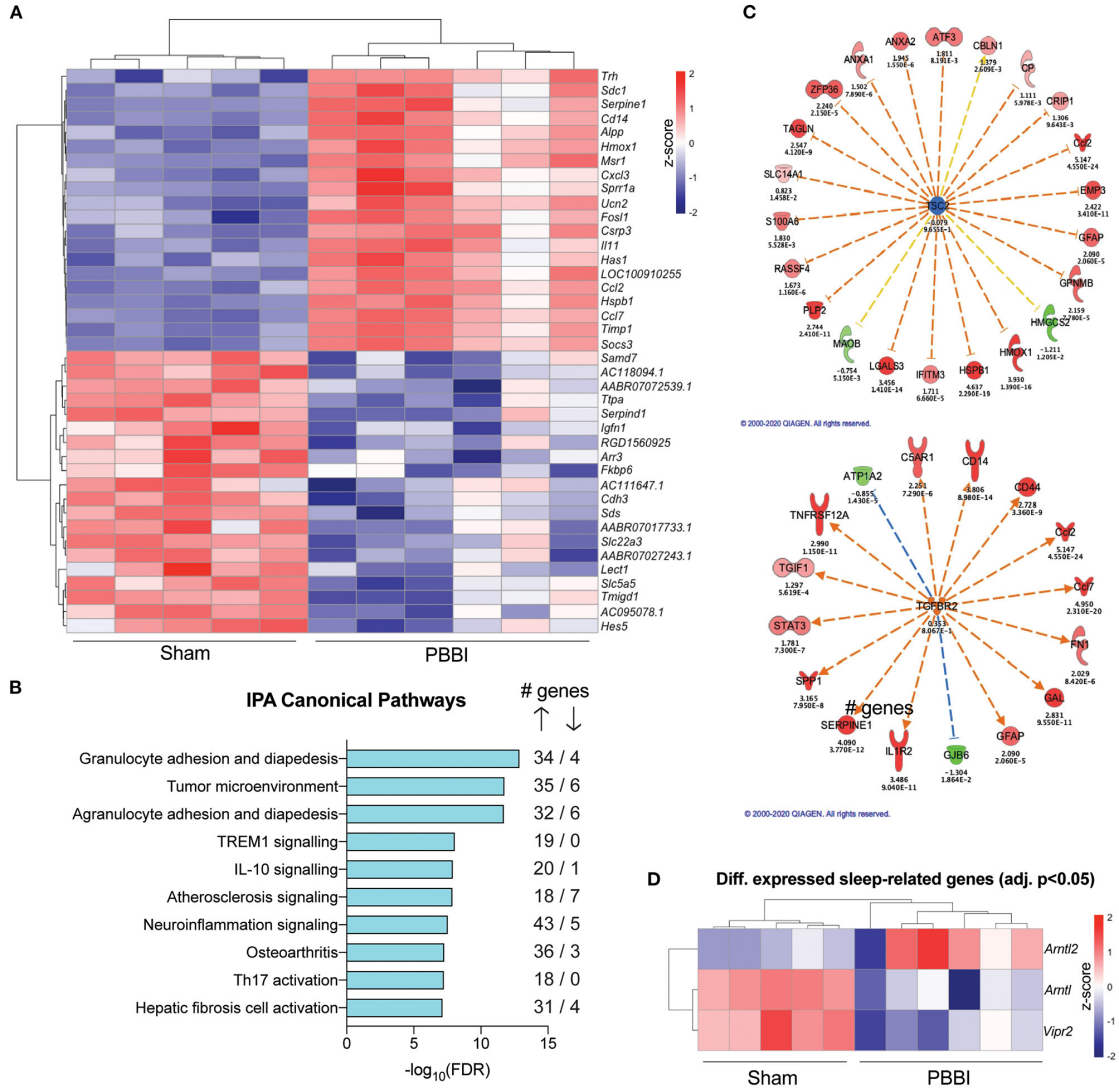
Effects of PBBI on transcriptomic networks for the injured hemisphere. **(A)** Top 20 (highest fold change in PBBI vs. sham) and bottom 20 (lowest fold change in PBBI vs. sham) genes that were differentially expressed between PBBI and sham in the ipsilateral hemisphere. Data are shown as z-scores for each individual subjects within each group and hierarchical clustering was performed. **(B)** Top ten canonical pathways identified using IPA in the ipsilateral hemisphere for PBBI vs. sham cortex. **(C)** Among the 1,755 differentially expressed genes within the injured cortex, there is significant enrichment of 2 upstream regulators, *TSC2* (left) and *TGFBR2* (right). Genes surrounding upstream regulators are as follows: Red molecule represents increased expression in PBBI vs. sham, while green represents decreased expression. Intensity of color represents amplitude of change. Orange dashed arrow represents predicted activation and blue dashed arrow represents predicted inhibition via the upstream regulator, while yellow dashed arrows represent inconsistent findings of activation using IPA's knowledge base. Significance is denoted by *p*-values below each downstream gene calculated by IPA. **(D)** Differentially expressed sleep-related genes in the ipsilateral hemisphere in PBBI rats vs. sham, shown as individual z-scores for each subjects within each group.

To identify differentially expressed genes following PBBI in the rodent cortex, we used next-generation RNA sequencing in cortical tissue from the hemispheres ipsilateral and contralateral to injury, due to the widespread effects of the PBBI across brain hemispheres (33). In the hemisphere ipsilateral to injury, gene expression profiling identified 1,755 differentially expressed genes between PBBI and sham groups (B-H corrected *p* ≤ 0.05; Supplementary Table 5). Of those, 1,339 had significantly increased expression while 436 genes were downregulated after injury compared to sham (Figure 7A). Using Ingenuity Pathway Analysis (IPA) software, we identified enriched canonical pathways within our data for up- and downregulated genes (log2FC >0.5 and < -0.5, adj. *p* < 0.05). Within this analysis, IPA's knowledge base identified the top ten canonical pathways as mainly those involved in immune regulation, including granulocyte adhesion and diapedesis, TREM1 and IL-10 signalling, and neuroinflammation signalling (Figure 7B). Specifically, the majority of genes within the top ten pathways were upregulated genes (89%) while the rest (11%) were downregulated, demonstrating the strength of the transcriptomic signature of immune activation following PBBI.

We also used IPA to ascertain upstream regulators of genes enriched in the PBBI group vs. sham and identified two main genes involved in downstream regulation of genes in the injured rat cortex: *Tuberous sclerosis complex 2 (Tsc2)* and *Transforming growth factor beta receptor 2 (Tgfbr2)*, both of which have been previously implicated in immune function and brain injury (44–46). 19 out of 22 genes in our dataset have a directional change consistent with inhibition of *Tsc2*, while 16 out of 16 genes in our dataset have a measurement direction consistent with activation of *Tgfbr2* (Figure 7C).

As the focus of this study was linking transcriptional outcomes to sleep parameters in our rodent model of PBBI, we also identified several genes critical to maintaining circadian rhythmicity that were altered following PBBI in the ipsilateral hemisphere, including *Arntl* (*Bmal1*; log2FC=-0.651, adj. *p* = 0.001), *Arntl2* (*Bmal2*; log2FC = 1.749, *p* = 0.003), and *Vipr2* (log2FC=-1.23, *p* = 1.9e-05) (Figure 7D). Finally, we also confirmed that, as expected, there were no statistically significant differences in gene expression between PBBI (*n* = 6) and sham (*n* = 5) cortex in the hemisphere contralateral to injury.

### Sleep-Associated Differential Gene Expression in the Cortex

**Figure 8:**
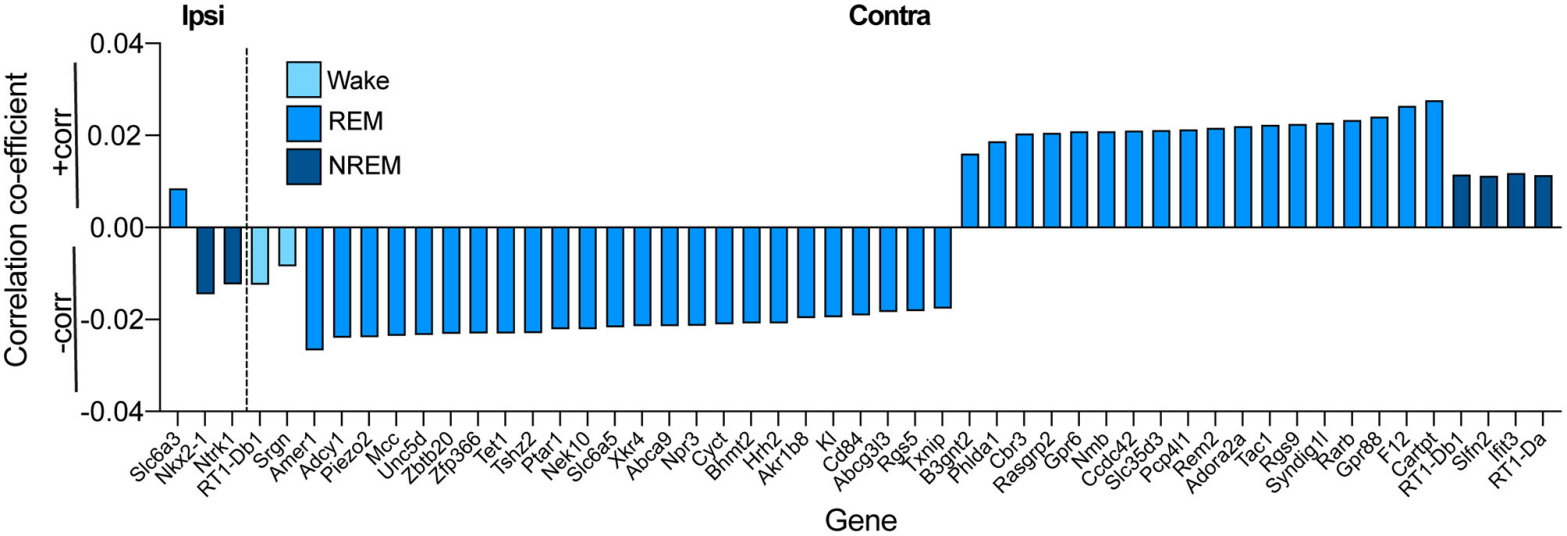
Association of time spent in sleep states vs. gene expression on the ipsilateral and contralateral sides of injury in both PBBI and sham cortex (conditions pooled together and injury was controlled for in statistical model) identified differentially expressed genes (adj. *p* < 0.05) associated with the three vigilance states examined. Directionality of the correlation between transcription and sleep are indicated as +corr (>0; positive correlation) or –corr (<0; negative correlation).

Due to the dramatic changes in sleep architecture in injured animals, we then sought to determine if there were differentially expressed genes in the ipsilateral and contralateral hemisphere depending on time spent in each sleep stage for all animals (both PBBI and sham animals; significantly altered genes shown in Supplementary Table 6). Injury was included in the statistical model to eliminate effects of injury on gene expression and focus solely on sleep parameters. In the ipsilateral hemisphere, we identified no differentially expressed genes associated with wakefulness after multiple testing correction, but we identified one gene significantly upregulated with increased time in REM sleep in the injured hemisphere, *Slc6a3*. Additionally, we identified two genes downregulated when increased time was spent in NREM sleep, *Nkx2-1* and *Ntrk1* (Figure 8). For the hemisphere contralateral to injury, we identified two genes downregulated when increased time was spent in wakefulness and four genes upregulated when increased time was spent in NREM. Interestingly, we found 24 genes downregulated and 18 genes upregulated when increased time was spent in REM (Figure 8).

## Discussion

We show here that a rodent model of PBBI, a severe penetrating traumatic brain injury, was associated with acute changes in sleep architecture and transcriptomic outcomes in the adult cortex. These changes were observed in pathways not only related to injury response such as inflammation and apoptosis, but also affected genes that have been implicated in the regulation of circadian rhythmicity and sleep. Unsurprisingly, we found that PBBI was associated with a massive upregulation of immune-related genes in the injured cortical hemisphere, including *Sprr1a, Cxcl3, Serpine1, Timp1, Ucn2, Il11, Csrp3, Hspb1, Ccl7, and Ccl2*. This was expected due to the nature and severity of the PBBI injury, and previous work has likewise shown immense immune activation after TBI (47–49) and especially PBBI (25, 50, 51).

We also identified two main hub genes, *Tsc2* and *Tgfbr2*, which were related to large networks of immune signaling genes that were altered in PBBI. These hub genes have previously been implicated in brain trauma and sleep architecture. For example, *Tgfbr2*, which codes for the receptor for TGF-B, an important immune marker (45), was upregulated in the cortex in response to cryogenic brain injury (52) and in the hippocampus after controlled cortical impact (CCI) brain injury (44) in rodents. Further, humans with a mutation in *TGFBR2* exhibit Loeys Dietz syndrome, of which a main symptom is sleep apnea (53). The other hub gene, *Tsc2*, has also been linked to circadian abnormalities and sleep dysfunction related to overactivity of mTOR signaling induced by knockout of *Tsc2*, likely due to its interactions with *Bmal1*, a well-established circadian regulator gene that is essential for maintaining circadian rhythms (54, 55). While these hub genes have clearly been implicated in sleep-related disorders, we also identified some differentially expressed genes in the PBBI group that are critical to maintaining circadian rhythmicity. For example, *Arntl/Bmal1* forms a heterodimer with another circadian gene, *Clock*, and this heterodimer binds to an E-box element causing increased expression of other circadian genes (Per1, Per2, Cry1, and Cry2). Knockout of the *Bmal1* gene has been shown to result in circadian arrhythmicity (55), as previously discussed. We observed a transcriptional increase of the *Bmal1* paralog, *Bmal2*. It has been shown that in the absence of *Bmal1, Bmal2* is capable of rescuing the clock and metabolic phenotypes of *Bmal1*-knockout mice, including rhythmic locomotor activity, rhythmic metabolism, low body weight, and enhanced fat deposition cite (56). We also noted a decrease in *Vipr2* transcription, the receptor for the neuropeptide vasointestinal peptide. The Vip/cAMP signaling regulates the long-term firing rate of neurons of the circadian pacemaker located in suprachiasmatic nucleus (SCN) of the hypothalamus (57), possibly via fast delayed rectifier (FDR) potassium currents (58).

In addition to changes in gene regulation relating to sleep, we directly measured sleep architecture using EEG monitoring on the ipsilateral and contralateral side of PBBI. PBBI animals had considerably altered sleep architecture and spent more time in NREM and less time in REM sleep and in Wake than sham animals which was evident from recordings both hemispheres. Other groups have likewise found impairments in sleep architecture after other rodent TBI models (59–65) [For a comprehensive review, see (15)]. Our findings of increased NREM and decreased wakefulness are in line with previous studies which used other rodent models of TBI, including controlled cortical impact (CCI) (60) and lateral fluid percussion (61). Dramatic changes in REM sleep after TBI vary in different rodent TBI models. While some studies (59, 60) did not find dramatic changes in REM sleep, a recent study by Kinduru et al. (66) using a controlled cortical impact (CCI) model in mice showed ~50% reduction in REM sleep acutely following injury. This suggests that the dramatic decrease in REM sleep produced by PBBI may be specific to this severe model of TBI and not present in non-penetrating rodent models. We then linked time in each sleep state with transcriptional outcomes and identified differential regulation of various genes depending on duration of each sleep state. Specifically, two genes, *Nk2 homeobox 1* (*Nkx2-1)* and *Neurotrophic receptor tyrosine kinase 1* (*Ntrk1 or TrkA)*, were downregulated in the injured hemisphere with increasing time spent in NREM. Knockdown of *Nkx2-1* has previously been linked to neurodegeneration and impaired sleep quality (67, 68), and *Nkx2-1* expression is altered after immune activation (69). Our findings were also in line with multiple groups that have shown that *Ntrk1* (also known as *TrkA*) is crucial for proper sleep architecture (70), and additionally that *Ntrk1* and its ligand *NGF* are directly altered following rodent models of TBI (71). We also found that a significant increase in expression of *Slc6a3* in the injured hemisphere was associated with increased time in REM sleep. This gene, which codes for the dopamine transporter or DAT1, has been associated with sleep architecture in large-scale studies of human participants (72–75), such that functional polymorphisms of the *DAT1* gene affected sleep duration and neurophysiological markers of sleep homeostasis. Further, DAT1 knockout mice display decreased hippocampal theta rhythms during wakefulness and REM sleep (76). DAT1 and dopamine release itself have also been altered following rat models of TBI and clinical cases (77). Taken together, our findings suggest that multiple genes that have been associated with sleep architecture show direct correlation to time spent in the sleep stage after PBBI in the injured hemisphere. We did find multiple genes in the contralateral (uninjured) hemisphere that showed a significant link to time in each sleep stage, with an abundance of genes associated with time in REM sleep, suggesting that effects on sleep, especially REM, may be mediated by indirect changes of gene expression in the contralateral hemisphere as well, although further research will identify how this occurs.

While this study provides novel insights into sleep-related gene expression resulting from PBBI in rodents, there were limitations to the current study. Primarily, the current study employed a small sample size (*n* = 5-6/group), which likely limited the statistical power and extension of conclusions regarding injury-induced spectral changes and sleep architecture. Additionally, sleep profiling and tissue extraction was limited to the acute (24 h) post-injury period; the timeline for resolution of both sleep architecture and gene expression remains to be determined. Future studies with additional samples and time points would aid in further conclusions regarding sleep physiology and neural gene expression. It is also important to note the correlational nature of gene expression changes with sleep architecture in this study, motivating future research to identify causative mechanisms behind PBBI-induced sleep changes and changes to gene expression. The intriguing results of these initial data warrant a comprehensive longitudinal analysis of disruptions in sleep-wake architecture in the PBBI model, as long-term changes in sleep are a common complaint following TBI in the clinical population (12). Studies to determine sustained changes in post-injury vigilance states, modified injury-induced EEG spectra, and prolonged frequency shifts are currently ongoing. Additionally, studies are ongoing to ascertain the protracted relationship between sleep-wake changes, lesion progression, and changes in circadian gene regulation outlined in these studies.

These data, although small in scope, demonstrate that the physiological response to a severe traumatic brain injury, like PBBI, is associated with acute changes in gene transcription that are further linked to PBBI-associated changes in sleep architecture. While acute response pathways such as inflammation, autophagy, and apoptosis typically follow major trauma, injury can also result in chronic neurodegeneration and aberrant behavior and cognition. Furthermore, injury-related changes in sleep may further exacerbate these pathways with long term negative health outcomes. Novel treatment of TBI may need to reflect the long-term temporal dynamics of the mechanisms regulating the target genes identified in this study. Thus, subsequent work should longitudinally investigate how methylation and transcriptional changes within these pathways are maintained and also replicate current findings with additional animals.

## Data Availability Statement

The datasets presented in this study can be found in online repositories. The names of the repository/repositories and accession number(s) can be found below: https://www.ncbi.nlm.nih.gov/geo/, GSE182360.

## Ethics Statement

The animal study was reviewed and approved by WRAIR IACUC. Material has been reviewed by the Walter Reed Army Institute of Research. There is no objection to its presentation and/or publication. The opinions or assertions contained herein are the private views of the author, and are not to be construed as official, or as reflecting true views of the Department of the Army or the Department of Defense. Research was conducted under an approved animal use protocol in an AAALAC International-accredited facility in compliance with the Animal Welfare Act and all other federal statutes and regulations relating to animals and experiments involving animals, and adheres to principles stated in the *Guide for Care and Use of Laboratory Animals*, NRC Publication, 2011 edition.

## Author Contributions

AM and DS designed the sleep experiments. MU performed tissue processing/RNA isolation. AM, WF, and JD carried out the injury model and sleep recordings and analysis. AM, ZW, YG, JB, and FH analyzed and interpreted RNAseq data and prepared the manuscript. All authors contributed to the article and approved the submitted version.

## Funding

This work was supported in part by the Veterans Affairs Office of Research and Development. FH is the recipient of a Research Career Scientist Award (#1IK6CX002074) from the United States Department of Veterans Affairs. FH research is supported by Veterans Affairs Merit Grants RX001705, CX001395, BX003794, and CX001728. This research was also supported by The Department of Defense, United States Army grant: H_014_2014_WRAIR.

## Conflict of Interest

The authors declare that the research was conducted in the absence of any commercial or financial relationships that could be construed as a potential conflict of interest.

## Publisher's Note

All claims expressed in this article are solely those of the authors and do not necessarily represent those of their affiliated organizations, or those of the publisher, the editors and the reviewers. Any product that may be evaluated in this article, or claim that may be made by its manufacturer, is not guaranteed or endorsed by the publisher.
